# Supplementary figures and images for: Mechanistic insights into the phosphoryl transfer reaction in cyclin-dependent kinase 2: A QM/MM study
Source: PLoS One. 2019 Sep 4;14(9):e0215793. doi: 10.1371/journal.pone.0215793 (PMC6726203; doi:10.1371/journal.pone.0215793)

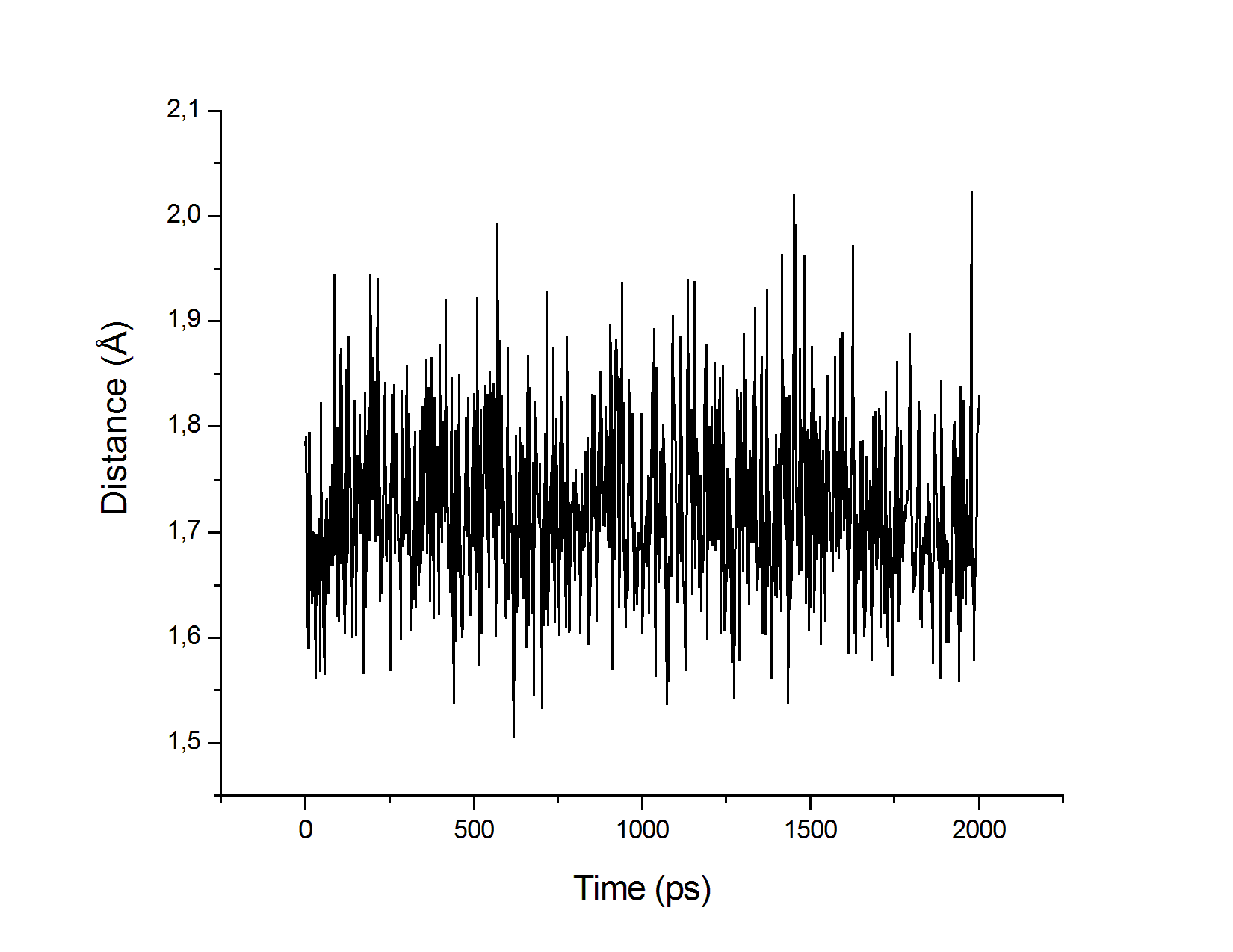

Supplement: S1 Fig — (PNG) [file pone.0215793.s001.PNG]
