# Supplementary material for: Mechanistic insights into the phosphoryl transfer reaction in cyclin-dependent kinase 2: A QM/MM study
Source: PLoS One. 2019 Sep 4;14(9):e0215793. doi: 10.1371/journal.pone.0215793 (PMC6726203; doi:10.1371/journal.pone.0215793)
Supplement: S1 Table — a reference [63]. b reference [64]. (DOCX) [file pone.0215793.s002.docx]

| **Structure** | **Calculated pK_a_** | **Experimental pK_a_** |
| --- | --- | --- |
| Asp127 | 3.5 | 3.6^a^ |
| Asp127 + active site (pSer excluded) | 3.9 |  |
| Asp127 + active site | 6.8 |  |
| pSer (O_2γ_) | 5.8 | 5.6^b^ |
| pSer (O_2γ_) + active site (Lys129 excluded) | 14.5 |  |
| pSer (O_2γ_) + active site | 20.2 |  |
| Lys129 | 10.2 | 10.5^a^ |
| Lys129 + active site (pSer excluded) | 4.3 |  |
| Lys129 + active site | 1.1 |  |
| Lys129 + active site (pSer optimized) | 10.9 |  |
