# Supplementary material for: Mechanistic insights into the phosphoryl transfer reaction in cyclin-dependent kinase 2: A QM/MM study
Source: PLoS One. 2019 Sep 4;14(9):e0215793. doi: 10.1371/journal.pone.0215793 (PMC6726203; doi:10.1371/journal.pone.0215793)
Supplement: S2 Table — (DOCX) [file pone.0215793.s003.docx]

| **Bond** | **Reac** | **TS** | **Prod** |
| --- | --- | --- | --- |
| O_γ_(Ser)-P_γ_ | 0.01 | 0.58 | 1.29 |
| O_3β_-P_γ_ | 0.76 | 0.03 | 0.00 |
| O_γ_(Ser)-H_γ_(Ser) | 1.24 | 0.64 | 0.00 |
| O_1γ_-H_γ_(Ser) | 0.15 | 0.69 | 1.20 |
