# Supplementary material for: Mechanistic insights into the phosphoryl transfer reaction in cyclin-dependent kinase 2: A QM/MM study
Source: PLoS One. 2019 Sep 4;14(9):e0215793. doi: 10.1371/journal.pone.0215793 (PMC6726203; doi:10.1371/journal.pone.0215793)
Supplement: S3 Table — (DOCX) [file pone.0215793.s004.docx]

| **Bond** | **Reac** | **TS1** | **Int** | **TS2** | **Prod** |
| --- | --- | --- | --- | --- | --- |
| O_γ_(Ser)-P_γ_ | 0.02 | 0.22 | 0.46 | 0.83 | 1.14 |
| O_3β_-P_γ_ | 0.78 | 0.12 | 0.03 | 0.00 | 0.00 |
| O_γ_(Ser)-H_γ_(Ser) | 1.32 | 1.25 | 1.17 | 0.70 | 0.13 |
| O_δ1_(Asp127)-H_γ_(Ser) | 0.10 | 0.13 | 0.17 | 0.62 | 1.21 |
| N_ζ1_-H_ζ1_ | 1.27 | 1.28 | 1.24 | 0.56 | 0.30 |
| O_2γ_-H_ζ1_ | 0.17 | 0.18 | 0.22 | 0.87 | 1.11 |
