# Supplementary material for: Mechanistic insights into the phosphoryl transfer reaction in cyclin-dependent kinase 2: A QM/MM study
Source: PLoS One. 2019 Sep 4;14(9):e0215793. doi: 10.1371/journal.pone.0215793 (PMC6726203; doi:10.1371/journal.pone.0215793)
Supplement: S4 Table — (DOCX) [file pone.0215793.s005.docx]

|  | **Substrate-assisted** | | | **Base-assisted** | | | | |
| --- | --- | --- | --- | --- | --- | --- | --- | --- |
| Atom | Reac | TS | Prod | Reac | TS1 | Int | TS2 | Prod |
| P_γ_ | 2.55 | 2.52 | 2.61 | 2.61 | 2.57 | 2.56 | 2.59 | 2.60 |
| O_3γ_ | -1.22 | -1.17 | -1.24 | -1.30 | -1.22 | -1.23 | -1.23 | -1.26 |
| O_2γ_ | -1.25 | -1.15 | -1.20 | -1.23 | -1.17 | -1.17 | -1.10 | -1.09 |
| O_1γ_ | -1.14 | -1.07 | -1.05 | -1.19 | -1.15 | -1.17 | -1.19 | -1.22 |
| PO_3_ | -1.07 | -0.87 | -0.89 | -1.12 | -0.98 | -1.03 | -0.94 | -0.97 |
| Mg | 1.47 | 1.50 | 1.51 | 1.44 | 1.45 | 1.47 | 1.50 | 1.51 |
