# Supplementary material for: Mechanistic insights into the phosphoryl transfer reaction in cyclin-dependent kinase 2: A QM/MM study
Source: PLoS One. 2019 Sep 4;14(9):e0215793. doi: 10.1371/journal.pone.0215793 (PMC6726203; doi:10.1371/journal.pone.0215793)
Supplement: S1 Protocol — (DOCX) [file pone.0215793.s006.docx]

**S1 Protocol**

**pK_a_ calculations**

Calculations were performed using the pK_a_ prediction module of Jaguar 10.4 [1,2] following the protocol of previous studies [3]. These are based on the following closed thermodynamic cycle:


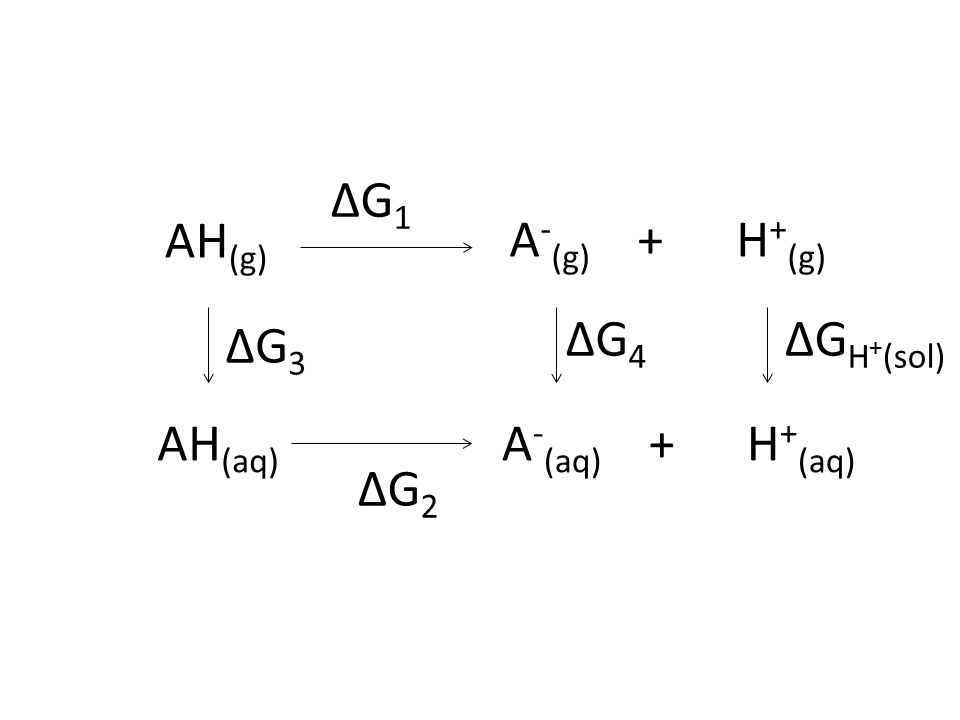


AH represents the protonated structure and A^-^ the deprotonated one. The pK_a_ is then calculated as [1]:

$pK_{a}=\frac{{\Delta G}_{2}}{2.3RT}=\frac{{\Delta G}_{1}+ {\Delta G}_{4}+{\Delta G}_{H^{+}\left( \mathrm{sol} \right)}-{\Delta G}_{3}}{2.3RT}$ (1)

${\Delta G}_{1}= \Delta H_{(g)}-T\Delta S_{\left( g \right)}=E_{A^{-}\left( g \right)}-E_{\mathrm{AH}\left( g \right)}+\frac{5}{2}RT-T\Delta S_{(g)}$ (2)

${\Delta G}_{3}= \Delta G_{AH(sol)}$ (3)

${\Delta G}_{4}= \Delta G_{A^{-}(sol)}$ (4)

A value of -259.5 kcal/mol was used for ${\Delta G}_{H^{+}\left( \mathrm{sol} \right)}$ [4] and $T\Delta S_{(g)}$ was approximated to a value of 7.8 kcal/mol, corresponding to the entropy of a proton [5]. $\frac{5}{2}\mathrm{RT}$ is the ideal gas approximation of the enthalpic contribution of a proton, where it is assumed the cancellation of equivalent terms for AH and A^-^. Cancellation of the entropic term for AH and A^-^ is also assumed. Thus, a “raw” pK_a_ can be obtained, however the protocol implements an empirical correction with two paramerters A and B that corrects for many of the approximations made, such as differences in zero point energies between AH and A^-^, errors in gas phase deprotonation energies, solvation energies, first shell hydrogen bonding interactions that are not trustworthy from the continuum electrostatic model, etc. Empirical corrections have been developed by comparing with experimental pK_a_’s values for a wide range of functional groups. The final pK_a_ value is then obtained via a simple linear fit:

$pK_{a}^{\mathrm{final}}=A(\mathrm{pK}_{a}^{\mathrm{raw}})+B$ (5)

Geometry optimizations of protonated and deprotonated forms are carried out at the level B3LYP/6-31G*, and single point calculations use the larger basis set cc-pVTZ(+) for the deprotonation step (A^-^ species) and cc-pVTZ [6] for the rest. Solvation free energies are computed using the self-consistent reaction field continuum solvation method [7] on the gas phase geometries. For more details of the protocol see reference [1].

Different QM (quantum mechanics) models were used to compute pK_a_ values which were based on the product complex conformation. The complete model included the active site residues Asp127, Lys129, Asn132, Asp145, Thr165, the Mg^2+^ ion and its coordinating water, the ADP molecule and the phosphorylated serine residue (pSer). The residues were cut between α and β carbons and were capped with hydrogen atoms. The ADP molecule is represented by the triphosphate moiety capped with a methyl group. All the residues included in the complete model were also part of the QM region in the QM/MM (quantum mechanics/molecular mechanics) calculations, with the exception of Thr165, which was included since forms a direct hydrogen bond with one of the side chain oxygens of Asp127, and therefore its inclusion is thought to be important for the calculation of the pK_a_. For the calculation of pK_a_ in Asp127, only the carboxyl group (COOH) was allowed to move while the rest of the residues were kept fixed during the optimization calculations of the protonated and deprotonated forms. For the calculation of pK_a_ in pSer at the O_2γ_ atom and Lys129, only the hydrogen atom at O_2γ_ and the NH_3_ group of Lys129 were allowed to move; this with the exception of one calculation where the phosphate group of pSer was also allowed to be optimized in the protonated and deprotonated states of Lys129. In all of these calculations the rest of the residues’ positions were kept fixed to preserve the geometry of the product state.

An important approximation is the use of a continuum solvent approach (water) to treat the protein’s active site, however we expect this approximation to be not too dramatic since CDK2 presents the glycine-rich loop in an open conformation what allows the entrance of many water molecules in the active site increasing its expected dielectric constant.

[1] Klicić JJ, Friesner RA, Liu S-Y, Guida WC. Accurate Prediction of Acidity Constants in Aqueous Solution via Density Functional Theory and Self-Consistent Reaction Field Methods. J Phys Chem A 2002;106:1327–35. doi:10.1021/jp012533f.

[2] MS Jaguar, Schrödinger, LLC, New York, NY, 2019.

[3] Cross JB, Duca JS, Kaminski JJ, Madison VS. The Active Site of a Zinc-Dependent Metalloproteinase Influences the Computed pKa of Ligands Coordinated to the Catalytic Zinc Ion. J Am Chem Soc 2002;124:11004–7. doi:10.1021/ja0201810.

[4] Reiss H, Heller A. The absolute potential of the standard hydrogen electrode: a new estimate. J Phys Chem 1985;89:4207–13. doi:10.1021/j100266a013.

[5] Lim C, Bashford D, Karplus M. Absolute pKa calculations with continuum dielectric methods. J Phys Chem 1991;95:5610–20. doi:10.1021/j100167a045.

[6] Dunning TH. Gaussian basis sets for use in correlated molecular calculations. I. The atoms boron through neon and hydrogen. J Chem Phys 1989;90:1007–23. doi:10.1063/1.456153.

[7] Marten B, Kim K, Cortis C, Friesner RA, Murphy RB, Ringnalda MN, et al. New Model for Calculation of Solvation Free Energies:  Correction of Self-Consistent Reaction Field Continuum Dielectric Theory for Short-Range Hydrogen-Bonding Effects. J Phys Chem 1996;100:11775–88. doi:10.1021/jp953087x.
